# Discussion and Round Table Presentation

**DOI:** 10.1155/1991/83151

**Published:** 1991


*HPB Surgery*, 1991. Vol. 4, pp 39–47
Reprints available directly from the publisher
Photocopying permitted by license only

© 1991 Harwood Academic Publishers GmbH
Printed in the United Kingdom

### DISCUSSION AND ROUND TABLE PRESENTATION

*Chairman Terblanche:* Most of the discussion time will be spent reviewing the long-term management of patients after a variceal bleed. In the last 10 minutes we will attempt to place the role of prophylactic therapy in perspective.

In my view there are some end-stage patients who should not be subjected to either emergency or definitive treatment. Unfortunately they are frequently difficult to identify as even apparently poor risk patients may well improve their liver status if they recover from the bleeding episode. There is also evidence to suggest that the risk of recurrent bleeding and death rapidly diminishes as time passes after a bleeding episode, with this risk returning to baseline within two to three months[1]. I believe that continued non-specific medical management alone is an option that should be considered in some otherwise fit patients who survive a single episode of variceal bleeding. This would allow one to determine whether they fall into the 30% of so of patients who will not have a further bleed. However, I concede that the majority of patients who have had a variceal bleed do require further therapy to prevent the next variceal bleed.

All of the panelists have agreed that repeated injection sclerotherapy is the most widely used form of therapy and emphasised the need to identify those patients who should be considered as failures of sclerotherapy. Such patients should be subjected to a surgical salvage procedure, either a shunt or a devascularisation and transection operation. The views of the panel differ on the exact criteria to be used and which patients should be subjected to operation. Bornman first drew attention to the fact that two sclerotherapy failures during a single hospital admission for an acute variceal bleed was associated with a prohibitive mortality in Child's category B and C patients and that such patients should be subjected to early surgical therapy[2]. Will the panel please define the current role of sclerotherapy and how they would identify sclerotherapy failures.

*Panelist Paquet:* Concerning the chairman's first statement that there are some patients who should not be subjected to either emergency or definitive treatment, I propose that emergency and definitive treatment should be separated. From my point of view, every patient with uncontrollable variceal haemorrhage needs emergency treatment as early as possible. This immediate endoscopic sclerosis should preferably be performed in centers with expertise. Otherwise, the patients should be transferred to such a center with a balloon tube in place. With this therapeutic strategy the results of emergency treatment of bleeding oesophageal varices using oesophageal balloon tamponade or immediate endoscopic injection sclerosis can be improved as has been demonstrated by our group and others[3–5]. If emergency injection sclerotherapy is not successful, patients should immediately undergo an emergency devascularisation procedure particularly if they are in the Child C group.

Since 1975 we have taken two early or long-term recurrences of oesophageal variceal hemorrhage or one recurrence of gastric variceal bleeding as an indication for surgical procedure. We try to stop oesophageal variceal bleeding again a second time by emergency sclerotherapy. In Child A and B patients we prefer a distal splenorenal or a narrow lumen mesocaval interposition shunt one to three weeks later. Additional selection criteria are liver volume (1000–2500ml) measured sonographically; portal perfusion (more than 10%) investigated by sequential scintigraphy; and exclusion of activity or progression of the liver cirrhosis or a stenosis of the arterial supply to the liver by laparoscopy and angiography respectively. We have demonstrated that these shunt procedures are the best form of treatment for bleeding oesophagogastric varices resistant to early and long-term endoscopic sclerotherapy[6].

*Panelist Henderson:* Sclerotherapy is the first line management for acute variceal bleeding, and ideally should be performed at the time of the first diagnostic endoscopy. Its role in the prevention of recurrent bleeding is less clear. Current data show sclerotherapy to have equal efficacy with beta-blockade, an overall long-term rebleeding rate in excess of 50%, and a "failure rate" of 25% – 30%. I would define sclerotherapy failure as rebleeding which cannot be controlled by repeated sclerotherapy. Rather than a set number of bleeding episodes or units of blood, the overall bleeding pattern should be considered. Three minor bleeding episodes over 3 years may be acceptable, while one massive rebleed from gastric varices is clearly a failure. In summary, I would advocate chronic sclerotherapy as the initial therapy to prevent rebleeding and use the time it buys to define the severity of the underlying liver disease.

*Panelist Idezuki:* It is true that sclerotherapy has become the first choice treatment for control of acute bleeding from varices in many institutions. In some of the patients varices disappear completely after a few sclerotherapy treatment sessions and never recur. However, it is also true that in many of the patients varices and bleeding do recur after this modality of treatment. From our experience, we feel that if the initial 3 to 4 sclerotherapy treatments fail to improve the grade of varices to red-color sign negative and $F_1$, it is often difficult to eradicate varices by sclerotherapy even if we continue further sessions of sclerotherapy. In these patients, we recommend operative treatment; that is oesophageal transection and extensive devascularisation with splenectomy in our department.

*Panelist Bornman:* Sclerotherapy is currently our first line of therapy for all patients admitted with proven oesophageal variceal bleeding. Only failures of sclerotherapy during the acute phase, as defined by two recurrent bleeding episodes during the same admission, are considered for salvage surgery. We prefer to do a simple transection of the oesophagus using an EEA staple gun. If this is not technically feasible, a standard end-to-side portacaval shunt or an eight millimeter interposition Gortex graft (Sarfeh Shunt) would be our second choice.

Long-term sclerotherapy failures are more difficult to define. The number of injection sessions and the duration taken to eradicate varices are not an accurate measure of success and even patients with persistent small varices often do well with no further bleeding. We are more concerned about patients who develop recurrent life-threatening bleeds during the phase before varices are eradicated or when this occurs with recurrent varices after initial successful variceal obliteration.

So far, we have been unable to identify patients who will respond poorly to long-term repeated sclerotherapy.

*Chairman Terblanche:* Thank you. That gives us a reasonable consensus, although there are some differences of opinion. If I can summarise: repeated injection sclerotherapy is the most widely used treatment in the initial long-term management of patients after a proven variceal bleed, with some form of surgery being reserved as a salvage procedure. Dr Henderson, you indicated in your presentation that the alternative to repeated injection sclerotherapy is long-term pharmacological therapy with beta blockade. I believe that this is still controversial and at the investigative level. The problems with beta blockade with propranolol include the need for continued long-term therapy with more than one dose a day, as well as the highly unlikely situation of obtaining complete patient compliance, particularly in alcoholic cirrhotic patients. There is some evidence to suggest that propranolol therapy might be helpful in the early phase after a variceal bleed to reduce recurrent bleeding while varices are being eradicated with sclerotherapy. An even more intriguing suggestion comes from a Danish group who propose that the combination of propranolol with sclerotherapy leads to more effective eradication of varices. They have shown that, in the short term, this combination reduces recurrence of oesophageal varices and bleeding after variceal obliteration[7]. There is also some evidence to suggest rebound bleeding may occur in patients who stop taking propranolol. Dr Henderson, would you like to put the case for long-term medical therapy?

*Panelist Henderson:* I find myself in the unusual position of being a surgeon and advocating a nonsurgical therapy. But we cannot ignore the data. Portal hypertension can be reduced pharmacologically and beta-blockade is only the first of what I feel sure will be other effective pharmacological therapy. While the case has clearly been substantiated for the use of propranolol (or long acting nadolol) in the prophylaxis of variceal bleeding, the data are conflicting on the use of beta-blockade to prevent recurrent bleeding[8,9]. Eleven randomized trials comparing beta-blockers to controls have shown an overall significant reduction in rebleeding from 67% to 39%, but no significant effect on mortality. Five randomized trials comparing beta-blockers to sclerotherapy have shown a 48% overall rebleeding rate in both groups, and mortalities of 32% and 30% repectively. I believe that if we accept the efficacy of sclerotherapy as a therapeutic modality in prevention of rebleeding, we must also accept beta-blockade as equally good.

*Chariman Terblanche:* Any other panelist?

*Panelist Idezuki:* We have no experience with propranolol. However, measuring intravariceal pressure and blood flow during sclerotherapy, we have found that varices with low pressure and low flow are more easily eradicated by sclerotherapy than those with higher pressure and high flow. It is possible that the use of propranolol in conjunction with sclerotherapy may be helpful.

*Panelist Paquet:* Our group could not confirm the results of the Kings College group[10] that beta blocker therapy was effective. We used metipranolol instead of propranolol. Metipranolol was administered 3 times per day (10 – 20 mg) reducing the heart rate by at least 25%. We were not able to reduce the frequency of recurrence of haemorrhage when at least one phase of endoscopic sclerotherapy was completed and the varices were reduced in size and protected by scar tissue[11]. However, it must be underlined that the timing of the administration of beta blockers differed from that of the Kings College group. They administered the drug directly after the first session of sclerotherapy and continued treatment until the varices were eradicated. Our patients took the drug after at least one phase of sclerotherapy (usually three to five sessions) or sometimes even two phases of sclerotherapy were finished and while they were waiting for the subsequent endoscopic sclerotherapy, usually performed after three or four months.

*Chairman Terblanche:* I believe that in the future we may well have other forms of medical management where a single daily dose of a relatively safe agent may make long-term medical therapy more attractive than it is at present. Despite Dr Henderson's comments, I believe that long-term medical therapy should only be undertaken in an expert centre under careful control and guidance.

I did not hear any of the panelists present a case for utilizing major surgical procedures, such as shunts or transection and devascularisation operations, in all patients. If this is agreed could I ask each of the panelists to again shortly summarise how they set about selecting patients for their favourite procedure after sclerotherapy has failed. Each has indicated this briefly in their papers but I believe it would be useful to attempt to arrive at some consensus.

*Panelist Bornman:* We prefer a devascularisation and oesophageal transection procedure for sclerotherapy failures, but in some patients this is not possible because of extensive peri-oesophageal fibrosis from repeated injection sclerotherapy sessions. Therefore, all patients undergo portography prior to surgery, in case a shunt procedure is required. In such cases we prefer a selective shunt for good risk patients and a standard portacaval shunt for poor risk alcoholic cirrhotic patients. However, an appreciable number of our sclerotherapy failures have end-stage liver disease and in these patients active treatment is withdrawn.

*Panelist Paquet:* Again, I repeat that we define "sclerotherapy failure" as either two early or late recurrences of oesophageal variceal bleeding during the course of endoscopic sclerotherapy, or one recurrent haemorrhage from gastric varices. Our favoured surgical procedure during the emergency situation, particularly in Child C patients, is a gastro-oesophageal disconnection (with or without splenectomy or fundoplication) and in cases with portal pressure over 40 cm of water, an emergency mesocaval shunt. During the bleeding free interval a distal splenorenal or a narrow lumen mesocaval interposition shunt is performed according to the above-mentioned criteria in Child's A and B patients.

*Panelist Henderson:* I would emphasise that I believe the choice of surgical procedure when sclerotherapy fails is dictated by the acuteness of bleeding and the severity of the liver disease. In my repertoire: i) the occasional patient with massive continued bleeding despite sclerotherapy will receive a large bore mesocaval shunt: ii) my most common surgical rescue is distal splenorenal shunt, which I believe should be used if control of bleeding and maintenance of liver function are the goal:

iii) liver transplantation should be considered for suitable candidates with end-stage liver disease.

*Panelist Idezuki:* As I have already mentioned, transection and devascularisation procedures can be performed safely without significant mortality and morbidity in patients of Child A and B categories. So we recommend these major procedures for patients in these categories who are under the age of 65. However, we have now stopped operating on Child C patients, and patients complicated with unresectable hepatoma. Also, we recommend continued sclerotherapy if the patient's age is over 65 years.

*Chairman Terblanche:* Clearly, controlled trials are going to help us achieve better definitions of which patient should be subjected to either a shunt or a devascularisation and transection operation. May I remind you that the preliminary data presented by David Triger from the Multicentre United Kingdom trial has shown that surgery does not confer appreciable benefit when comparing oesophagogastric devascularisation and transection with endoscopic sclerotherapy in long-term management. Portacaval shunt trials have provided conflicting data. I accept the Atlanta proposal that sclerotherapy be the primary therapy with shunt salvage (or devascularisation and transection salvage) being used for those patients who are considered failures. Long-term treatment failure should be considered either when patients continue to have repeated bleeds despite sclerotherapy or when the oesophageal varices are difficult to eradicate. Major surgery, unfortunately, has to be restricted to the Child's A and B group patients and to the better risk Child's C patients. Poor risk end-stage Child's C patients should not be subjected to elective surgery, other than an hepatic transplant when indicated.

The use of narrow lumen shunts, either portacaval or mesocaval, has been alluded to by both Dr Paquet and Dr Idezuki. It was Henri Bismuth who first suggested narrowing the diameter of a shunt. The leader in narrow lumen shunts has been Sarfeh who has clearly emphasized the role of narrow diameter reinforced portacaval shunts. Would the other two panelists like to comment on their views on the current role of narrow lumen shunts combined with devascularisation and compartmentalisation operations?

*Panelist Henderson:* My conversion to accepting a potential role for narrow lumen shunts comes from data in the propranolol studies, which show that reduction of hepatic venous pressure gradient to 12 mm Hg or less prevents variceal bleeding. This figure is identical to that defined by Dr Sarfeh with an 8 mm shunt in his careful haeomodynamic studies. I think more data may support their use.

*Panelist Bornman:* The narrow lumen interposition shunt is an attractive option by virtue of its simplicity but we would consider this operation as a salvage procedure only at this stage during the acute bleeding episode. One would like to see more long-term patency results before advocating this as a definitive shunt procedure for good risk patients.

*Chairman Terblanche:* When we consider shunts in otherwise fit patients in 1990 then the main options are the distal splenorenal shunt or a narrow lumen shunt with disconnection. I note that there is agreement from all the panelists.

*Chairman Terblanche:* There appear to be several devascularisation and transection operations available for long-term management. In Cape Town we use a procedure similar to that described by Dr Paquet, namely, extensive upper gastric and lower oesophageal devascularisation with an oesophageal stapled transection without splenectomy. We are currently conducting a controlled trial comparing this treatment with sclerotherapy. We perform a highly selective vagotomy along the lesser curve of the stomach and disconnect the short gastric vessels from the spleen, leaving the spleen *in situ*, and then devascularise the lower 6 to 7 cm of the oesophagus prior to transecting the oesophagus. We currently combine this with a fundoplication. In some patients we have noted large perforating veins entering the oesophagus relatively high up and I believe that one needs to look for these perforators very carefully. Today, devascularisation without transection has been largely abandoned on the basis of the long-term results of the studies in patients, other than those with schistosomiasis. In patients who have had devascularisation and transection procedures, if varices recur, they can usually be relatively easily and successfully treated with subsequent sclerotherapy. The recurrence rate after devascularisation and transection operations appears to be higher than after shunting. Do any of the panelists wish to comment?

*Panelist Idezuki:* We are still doing extensive devascularisation without transection of the oesophagus in patients whose varices are limited to the region of cardia of the stomach and also in acute bleeders who fail with sclerotherapy treatment.

It is not safe to transect the oesophagus where the inflammatory process, which is caused by injection of sclerosants, is still active and in these cases we only perform extensive devascularisation and splenectomy. Residual varices after devascularisaion can usually be treated easily by sclerotherapy.

Regarding the recurrence rate of varices after transection and devascularisation, it may be slightly higher than after shunt operations but recurrent bleeding has been around 7% which we consider to be acceptable.

*Chairman Terblanche:* I believe that there is general consensus on the panel that hepatic transplantation should be considered in all patients who present with a variceal bleed but that only a very small percentage of our patients will ultimately qualify for a liver transplant. In those who are likely to be transplant recipients, sclerotherapy and/or drug therapy would be the initial treatment of choice. For the failures of sclerotherapy, a transection with or without devascularisation would be preferable to a shunt and if a shunt is performed, a narrow diameter reinforced PTFE mesocaval graft would be preferable to a portacaval shunt. Several groups have indicated that liver transplantation is perfectly feasible and can be performed with reasonable safety after a previous portacaval shunt, but this does increase the technical difficulty of the operation. Do the panelists agree?

*Panelist Henderson:* A potential liver transplant patient who fails sclerotherapy when their own liver still has 2 to 5 years of life in it should have a distal splenorenal shunt. A mesocaval shunt should be used as a life saving (stop bleeding) operation in a patient who may come to early transplant. These two procedures are more remote from the operative field for transplantation than a transection/devascularisation procedure. I do not personally favour the latter as I believe they make subsequent transplant more difficult.

*Chairman Terblanche:* I would now like to turn to prophylaxis or therapy in patients who have portal hypertension and varices but who have not yet had a variceal bleed. It would perhaps we helpful if I was to provide a brief overview of my own throughts and then ask the panelists to comment.

My views on prophylactic therapy have been clearly stated in the literature and have not changed in 1990[12]. Options in prophylactic therapy include the following. One is to undertake no specific therapy and wait until the first bleed occurs. This is the option that I believe should be followed in 1990 because it has been clearly demonstrated that the majority of patients do not bleed prior to their death from liver failure if they have not had a previous bleed. Estimates vary, but between 70% and 80% of patients will not bleed. Under these circumstances 70% to 80% of patients would be receiving any prophylactic therapy used unnecessarily. This is unjustified. The problem has been to identify patients at high risk of a variceal bleed and only subject them to prophylactic therapy. Both the Japanese group of Dr Idezuki and the German group of Dr Paquet appear to have identified a high risk group of patients, but I would like to hear their views. I believe that prophylactic therapy remains unjustified outside of major centers or controlled trials at this time. The most important information on the identification of high risk patients comes from the North Italian Endoscopic Club who have utilized three separate factors in formulating their index of a high probability of a first variceal bleed. These factors are the clinical Child's class of the patient; the size of the varices; and the presence of red wale markings. They have been able to identify a subset of patients at very high risk of bleeding[13]. If this is confirmed by other groups then some form of prophylaxis, particularly prophylactic drug therapy, may well become indicated. A recent meta-analysis from Edinburgh of the value of propranolol in the prevention of variceal haemorrhage comes out in favour of propranolol for primary prevention of variceal haemorrhage in patients with large varices[9].

*Panelist Henderson:* I believe that prophylactic propranolol therapy is justified in patients who have varices identified. The data persuade me that this will reduce the risk of their initial bleed by 50%. I agree that it may only be a delay, but I think it is justified and worthwhile.

Prophylactic sclerotherapy is not justified by current data. Better definition of higher risk groups, such as the work being done by NIEC may change this.

Surgical prophylaxis has resurfaced in 1990 with the Japanese multi-center study of portal nondecompressive surgery which shows a significant survival advantage to the operated group. I do not believe this should be generally advocated, but it does warrant further study.

*Panelist Paquet:* The aim of long-term prophylactic treatment of cirrhotics with oesophageal varices is to prevent the first episode of variceal haemorrhage and thereby improve survival. Only one-third of patients with cirrhosis and varices are thought to be likely to bleed. If all patients with cirrhosis and varices were treated prophylactically, two-thirds will be managed unnecessarily. Thus, prophylactic sclerotherapy is only justified in one-third of all cirrhotics with portal hypertension. Furthermore, in many studies the bleeding risk of this third can be estimated by the following risk factors: 1. Large varices (degree III to IV), according to our classification; 2. Appearance of the varices – telangiectasis or cherry red spots on varices and; 3. An intravariceal oesophageal or portal or wedged occluded hepatic pressure over 22 mmHg. In a prospective controlled randomized trial, 61 consecutive patients from a group of 232 were selected for prophylactic endoscopic sclerotherapy or as controls. If bleeding occurred in control patients, the therapy of choice was endoscopic sclerotherapy. The frequency of variceal haemorrhage in the control group was 74% (23 patients) and 30% (10 patients) in the sclerotherapy group. Death after a median follow up of 14 months was 62% (20 patients) in the controls and 20% (6 patients) in the sclerotherapy group. Thus sclerotherapy was more efficient in preventing first bleeding and prolonging survival in selected cirrhotic patients with a high risk of variceal haemorrhage, particularly Childs A and B patients. Only in this highly selected group is prophylactic endoscopic sclerotherapy justified.

*Panelist Idezuki:* I have already presented our data on prophylaxis in the paper. I would only like to point out that the number of patients undergoing prophylactic surgery in Japan has decreased considerably since the development of sclerotherapy, and now patients who have risky varices are treated by prophylactic sclerotherapy. We may concede that some of these patients may not have bled during their lifetime and prophylactic treatment may not have been necessary and that we are overtreating these patients. But at the same time no one can yet predict who is going to bleed and when it will happen. Some of these patients may die from the first bleeding episode, if the proper treatment is not available and initiated immediately. If we consider this risk and if we can treat varices with less invasive methods, without any risk, there may be a place for prophylactic treatment.

*Chairman Terblanche:* Thank you, What is your view Dr Bornman?

*Panelist Bornman:* I agree.

*Chairman Terblanche:* In concluding this symposium I would like to thank the panelists for their wonderful co-operation. I believe that we have been able to place the long-term management of varices in perspective, particularly in patients after a previous variceal bleed. We also appear to have reasonable consensus on the current situation with regard to prophylactic therapy. The audience might like to note that in the July 1990 issue of the American Journal of Surgery, our panelist Mike Henderson has assembled papers from most of the world's experts in a special commemorative issue dedicated to Dr W Dean Warren. Thank you all for your participation.

### *References*

1. Smith, J.L. and Graham, D.Y. (1982) Variceal hemorrhage: a critical evaluation of survival analysis. *Gastroenterology*, **82**, 968–973
2. Bornman, P.C., Terblanche, J., Kahn, D., Jonker, M.A. and Kirsch, R.E. (1986) Limitations of multiple injection sclerotherapy sessions for acute variceal bleeding. *South African Medical Journal*, **70**, 34–36
3. Paquet, K.-J. and Feussner, H. (1985) Endoscopic sclerosis and esophageal balloon tamponade in acute hemorrhage from esophagogastric varices: a prospective controlled randomized trial.*Hepatology*, **5**, 580–583
4. Paquet, K.-J., Kalk, J.-F. and Koussouris, P. (1988) Immediate endoscopic sclerosis of bleeding esophageal varices: a prospective evaluation over five years. *Surgical Endoscopy*, **2**, 18–23

5. Prindiville, T. and Trudeau, W. (1986) A comparison of immediate versus delayed endoscopic injection sclerosis of bleeding esophageal varices. *Gastrointestinal Endoscopy*, **32**, 285–288
6. Paquet, K.-J., Mercado,M.A. and Gad, H.A. (1990) Surgical procedures for bleeding esophago-gastric varices when sclerotherapy fails: a prospective study. *American Journal of Surgery*, **160**, 43–47
7. Jensen, L.S. and Krarup, N. (1990) Propranolol may prevent recurrence of oesophageal varices after obliteration by endoscopic sclerotherapy. *Scandinavian Journal of Gastroenterology,* **25**, 352–356
8. Grace, N.. (1990) A Hepatologist's view of variceal bleeding. *American Journal of Surgery*, **160**, 26–31
9. Hayes, P.C., Davis, J.M., Lewis, J.A. and Bouchier, I.A.D. (1990) Meta-analysis of value of propranolol in prevention of variceal haemorrhage. *Lancet*, **336**, 153–156
10. Westaby, D., Melia, W., Hegarty, J., Gimson, A.E.S., Stellon, A.J. and Williams, R. (1986) Use of propranolol to reduce the bleeding rate during injection sclerotherapy prior to variceal obliteration. *Hepatology*, **6**, 673–675
11. Paquet, K.-J. and Feussner, H. (1983) Ist der Betablocker Metipranolol zur Prophylkaxe von Blutungsrezidiven nach Wandsklerosierung der Speiserohre wegen blutenden Osophagusvarizen geeignet? *Z Gastroent*, **21**, 427 (Abstr)
12. Terblanche, J., Burroughs, A.K. and Hobbs, K.E.F. (1989) Controversies in the management of bleeding esophageal varices (Second of Two Parts). *New England Journal of Medicine*, **320**, 1469–1475
13. The North Italian Endoscopic Club for the Study and Treatment of Esophageal Varices. (1988) Prediction of the first variceal hemorrhage in patients with cirrhosis of the liver and esophageal varices: a prospective multicenter study. *New England Journal of Medicine*, **319**, 983–989

